# Wildfire: distributed, Grid-enabled workflow construction and execution

**DOI:** 10.1186/1471-2105-6-69

**Published:** 2005-03-24

**Authors:** Francis Tang, Ching Lian Chua, Liang-Yoong Ho, Yun Ping Lim, Praveen Issac, Arun Krishnan

**Affiliations:** 1 Information Science Research, Bioinformatics Institute, 30 Biopolis Street, #07-01, Matrix, 138671 Singapore; 2 Operations Research Lab, DSO National Laboratories, 20 Science Park Drive, 118230 Singapore; 3 Singapore Biomedical Computing Resource, Bioinformatics Institute, 30 Biopolis Street, #07-01, Matrix, 138671 Singapore; 4 Global Software Group, Motorola Electronics Pte Ltd, 12 Ang Mo Kio St. 64, Ang Mo Kio Industrial Park 3, 569088 Singapore

## Abstract

**Background:**

We observe two trends in bioinformatics: (i) analyses are increasing in complexity, often requiring several applications to be run as a workflow; and (ii) multiple CPU clusters and Grids are available to more scientists. The traditional solution to the problem of running workflows across multiple CPUs required programming, often in a scripting language such as perl. Programming places such solutions beyond the reach of many bioinformatics consumers.

**Results:**

We present Wildfire, a graphical user interface for constructing and running workflows. Wildfire borrows user interface features from Jemboss and adds a drag-and-drop interface allowing the user to compose EMBOSS (and other) programs into workflows. For execution, Wildfire uses GEL, the underlying workflow execution engine, which can exploit available parallelism on multiple CPU machines including Beowulf-class clusters and Grids.

**Conclusion:**

Wildfire simplifies the tasks of constructing and executing bioinformatics workflows.

## Background

Seemingly small steps in usability of bioinformatics applications have, perhaps, been the most important to the bioinformatics consumer. Suites such as Accelrys SeqWeb and EMBOSS/Jemboss [1,2], through consistent user interface elements, have narrowed the usability gap and made individual applications accessible to the non-specialist bioinformatician.

Bioinformatics analysis is becoming more complex, often requiring several applications to be run in combination in a workflow. Beowulf-class clusters have made supercomputing affordable, allowing us to execute workflows faster, including some which would previously have been infeasible. Traditionally, building such workflows required programming expertise, often in scripting languages such as perl. The usability gap in bioinformatics has now moved from individual applications to both construction and execution of workflows.

Existing efforts in narrowing this gap include Jemboss [2], Taverna/Freefluo [3], ICENI [4] and Biopipe [5]. Jemboss, though it does not support workflows directly, addresses usability of bioinformatics applications by providing a graphical user interface to EMBOSS. The user interface replaces the command-line options of the EMBOSS applications with interface elements such as check boxes, drop-down lists and text boxes. This simplifies the applications for users unfamiliar with command-line interfaces; even for command-line enthusiasts, it simplifies learning of new applications, which might only be used occasionally, since the interface is consistent across applications. Jemboss can run the EMBOSS application on the same machine ("Stand-alone mode") or remotely using a SOAP protocol.

Taverna, by default, constructs workflows which use Web-Services for components. The interface requires the user to connect together output and input ports of components to build a workflow. Taverna relies on Soaplab [6] to convert the EMBOSS command-line applications into Web-Services. However, Soaplab appears to have lost the help text annotations of the different input fields which is characteristic of other EMBOSS interfaces. ICENI is also a service-oriented workflow framework and has a Netbeans-based user interface.

Biopipe is a workflow framework which also allows for execution of workflows across clusters. However, Biopipe only allows for pipelines, not more general workflows with iterative loops; in particular, the particle swarm optimisation example presented later cannot be implemented in Biopipe. Also, Biopipe currently does not have a user-friendly interface for building pipelines.

In the next section, we introduce Wildfire which provides an integrated environment for construction and execution of workflows. It provides an intuitive interface based on a drawing analogy and, like Jemboss, presents program options using graphical user interface elements; thus Wildfire hides the precise syntax of scripting languages and command-line options from the user. Jemboss can run several independent processes in the background, but it has no dependency handling facility, whereas Wildfire allows the user to compose applications into a workflow. We illustrate by presenting some examples in the Results section. In contrast to Taverna and ICENI, Wildfire works directly with program executables, rather than Web- or Grid-Services. For execution, it uses GEL (Grid Execution Language [7]) which can run the workflow over the compute nodes of a cluster, similar to Biopipe. However, GEL can also run executables directly, or on the Grid. Thus, Wildfire and GEL bring supercomputing power to the bioinformatician.

## Implementation

**Figure 1:**
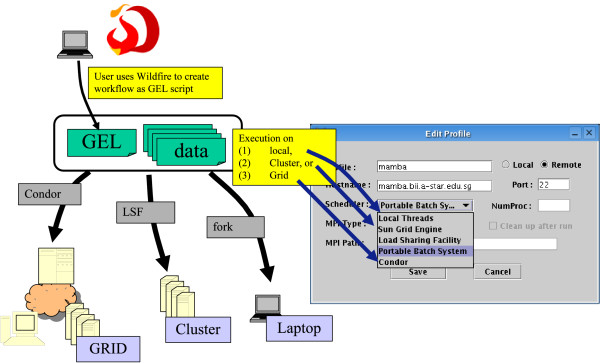
**Relationship between Wildfire and GEL**. Relationship between Wildfire and GEL. Wildfire is an interactive application which allows users to construct workflows using a drawing analogy. Wildfire executes the workflow by exporting it as a GEL script which is executed using a suitable GEL interpretor. There are GEL interpretors for execution on (i) the Grid, using Condor, (ii) a cluster, using LSF, PBS or SGE, and (iii) the same machine, which could be a laptop, desktop or multi-processor server.

Wildfire allows the user to visually construct workflows. For execution, Wildfire exports the workflow as a GEL script, and then calls a GEL interpretor to execute it. The GEL interpretor can either run on the same machine as Wildfire, or on a remote compute server. Figure 1 summarises the interaction between Wildfire and GEL.

Wildfire is implemented in Java, and has been tested on Windows and Linux platforms. On a Linux platform, the user can run workflows directly on the same machine: ideal for developing and testing small examples on a laptop, while reserving the multi-processor servers and clusters for running the workflow on real data.

We next describe the two main activities enabled by Wildfire: *construction *and *execution *of workflows.

### Workflow construction

When constructing workflows, the user does not need to work directly with the syntax of scripting languages such as GEL or perl. Rather, the user is presented with a graphical workflow canvas. On the canvas, a workflow component can be (i) an *atomic component*, (ii) a subworkflow or (iii) a loop (both parallel and sequential). An atomic component approximately corresponds to an EMBOSS application; in particular, each atomic component has an ACD (Ajax Command Definition [8]) description of its parameters and options. The user can select the atomic components from a customisable list of templates, which by default includes all the EMBOSS 2.8.0 applications (see Availability and requirements section). Components are visually rendered on the canvas as yellow rectangles labelled with the component name (e.g. EMBOSS program name), and a unique numerical identifier which can be used distinguish instances from the same component template. Sequential dependencies between components are created by drawing an arrow between them. By default, components not linked by arrows are assumed to be independent (and so can be executed in parallel).

**Figure 2:**
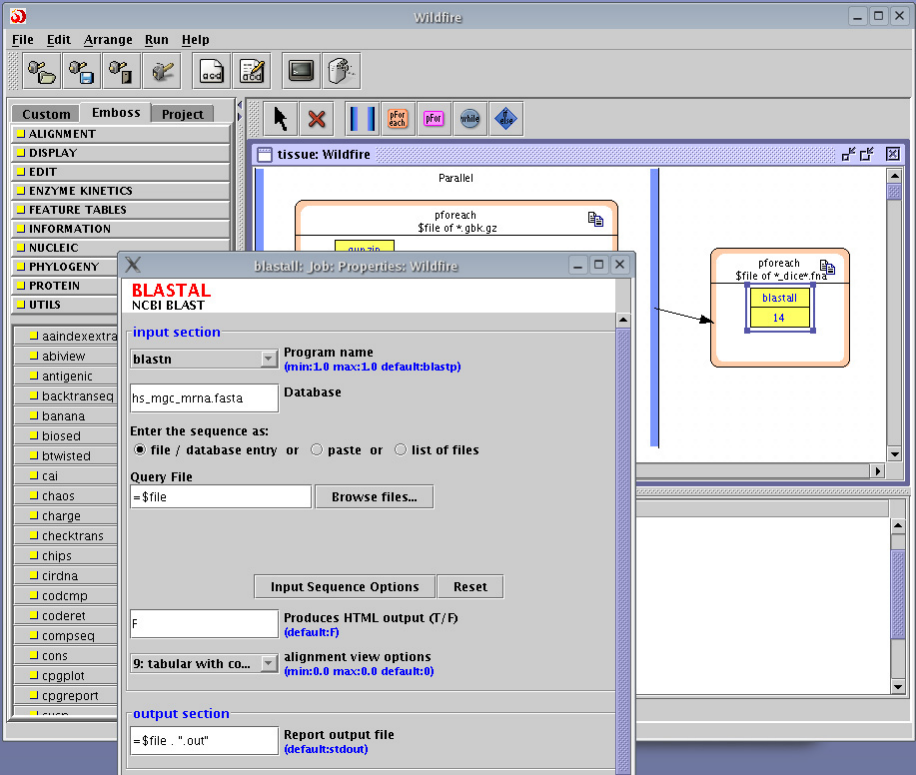
**Elements of the Wildfire interface**. Elements of the Wildfire interface. The main window in the background shows the workflow canvas. Its left panel lists the atomic workflow components; this list is preconfigured with all EMBOSS applications and can be customised. The foreground window shows the properties form for the blastall program.

Double clicking on an atomic component in the workflow will bring up a properties window resembling that of Jemboss (see Fig. 2). Wildfire uses Jemboss code to parse the ACD [8] description of the application to construct the form and provides default values where defined. These forms simplify configuration options by replacing the command-line flags and switches with graphical user interface elements such as drop-down menus. Help text annotations for the input fields save the user the effort of looking up UNIX man- or EMBOSS tfm-pages.

Wildfire extends the Jemboss interface by allowing the user to use expressions (similar to spreadsheet formulae) in the text fields. For example, in Fig. 2, the query file for blastall is = $flie. The first letter is an equals symbol (=) and indicates that this is not a literal string, but an expression. The remainder is the expression meaning "the value of variable $flie". Here the value of $flie is determined by the pforeach container, as shown in the background window, and denotes a parallel composition of blastall instances with $file set to the different files matching *_dice*.fna. The output file is

= $file . ".out"

which is an expression meaning "the value of variable $flie with .out appended". Another example of an expression is

= $f % ".fasta"

which means "the value of variable $f without the .fasta extension". The % and . operators can be mixed, for example

= $f % ".fasta" . ".pep"

which replaces the .fasta extension with .pep.

In addition, the user can add his/her own command-line programs to the list of atomic components by providing a description of its command-line options using an extended ACD syntax. The Wildfire user interface has a facility to help the user write ACD files for new atomic components. The interface shields the user from the complex ACD syntax.

Other than defining the dependencies between components and the invocation arguments, the user can place input files required by the workflow in subdirectories within the workflow directory. Wildfire can instruct GEL to copy files from these subdirectories into the working directory before a component is executed. Any instance can specify input files, thus allowing for files to be staged-in in a just-in-time manner. However, a common workflow pattern is one which specifies all input files to copied only by the first instance in the workflow.

### Workflow execution

For execution, Wildfire exports a programmatic description of the workflow, in a scripting language called GEL [7], which is passed to a GEL interpretor for execution. GEL is a scripting language with parallel constructs characterising common parallel workflow execution patterns. It is designed to be a generic parallel scripting language which can be executed on different types of homogeneous and heterogeneous parallel hardware such as shared-memory SMP servers, clusters with a shared disk image, and Grids without a shared disk image. There currently exist interpretors that can run GEL scripts on SMP servers, clusters with Platform LSF, PBS or Sun GridEngine (SGE), and on Condor Grids [9,10]. GEL is similar to APST [11], NIMROD [12] and DAGMan (part of Condor) but also allows for cyclic dependencies between jobs. The reader is referred to [7] for a more thorough description of GEL.

When developing small workflows, the user can run the workflow on the same machine (see Availability and requirements section). In this way, Wildfire can be used as a stand-alone application without access to the network.

**Figure 3:**
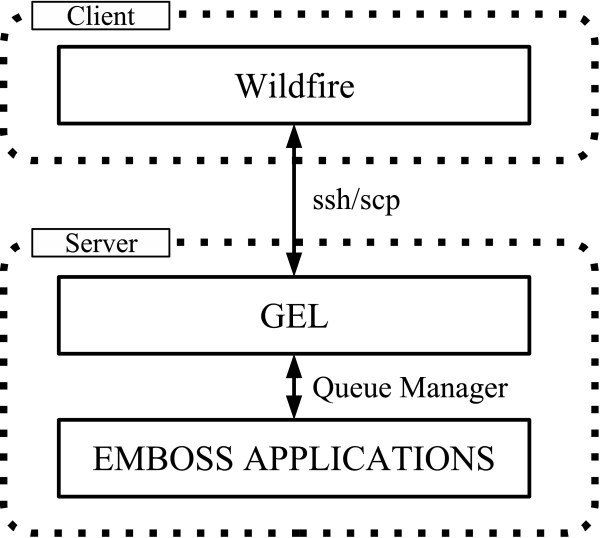
**Remote execution of workflows**. Remote execution of workflows. In the case of large workflows or when the applications are not available on the client machine, it is possible to execute the workflow remotely. In this case, Wildfire uses the secure shell (SSH) protocol to send the files to the remote machine and start execution. The remote machine can be a cluster or multiprocessor server.

Alternatively, the user can choose to send the workflow to a remote server and run it there. In this case, Wildfire uses the secure shell (SSH) protocol to send the necessary files over to, and then run the GEL interpretor on the remote server (see Fig. 3). The GEL interpretor can execute the atomic components directly if the server has multiple processors. If the server is a cluster, then GEL can submit the atomic components as jobs to the queue manager. In either case, the GEL interpretor will try to use multiple processors where possible. Remote server execution is useful for workflows with large data sets since GEL will make use of multiple processors. It is also useful if the atomic components are not installed on the local machine.

Wildfire and GEL do not require super-user privileges to install: they can be installed in the "home" directory. For the client-server mode of operation, only an SSH service on the server is required; there is no need to configure other services such as SOAP over HTTP or Web-/Grid-Services, and the firewall is only required to allow incoming SSH connections. Most modern UNIX-style configurations already provide an SSH service.

Wildfire can also use GEL to break up the workflow and run parts of it concurrently on different supercomputers using Condor. (Note: GEL 1.0 uses the Globus [13] protocols to provide Grid support. GEL 2.0 uses Condor for Grid execution and future support for Globus Grids will be via Condor-G.) This is useful for very large workflows which require as many compute resources as possible. In practice, it is more useful when not all components are available on any one machine, for example, because of licence availability.

Wildfire monitors execution of the individual atomic components and feedback is provided via annotations on the canvas which are updated in real-time.

The exported GEL script can also be run directly using an interpretor via the command line. This allows a workflow to be run in batch mode independently of Wildfire, and is useful for very long-running workflows or those that have to be run repeatedly.

## Results

We describe three applications of Wildfire. In the first application, we construct a workflow for analysis of human tissue-specific transcripts by comparing them against the known exons. This example shows how Wildfire can make use of the parallel capabilities of supercomputers. The second example considers a particle swarm optimisation algorithm implemented as a workflow, and shows that Wildfire can express workflows requiring iteration. The last example cross validates an allergenicity prediction algorithm. The number of parallel processes in this workflow can only be determined at run-time.

### Tissue-specific Gene Expression Analysis

To study tissue-specific gene expression in humans, we compare the known exons against a database of 16,385 transcripts obtained from the Mammalian Gene Collection. Since the human genome contains many exons, the extraction process is time consuming, but it is easily parallelised. The standard organisation of the 24 chromosomes into separate files provides a natural partitioning of the exon extraction problem: we extract the exons from each chromosome in parallel. To further increase the granularity of the problem and so exploit more parallelism, we break up each of the 24 files of exons into five smaller files, resulting in a total of 120 files. We blast each of these smaller files against the database of transcripts.

**Figure 4:**
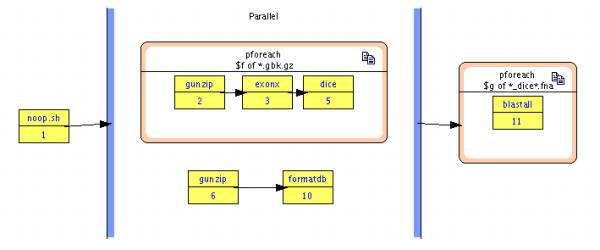
**Tissue-specific gene expression analysis**. Tissue-specific gene expression analysis. We initially start with 24 compressed genbank files, one for each human chromosome. We decompress (gunzip) the genbank file, extract (exonx) from it all the exons into one fasta file and then break up (dice) this fasta file into 5 smaller fasta files; we do this for each chromosome in parallel. At the same time, we decompress the file of transcripts and use formatdb to format it for use as a BLAST database. Finally, we blast the exons against the transcript database in parallel. Note that components are rendered as rectangles and the bottom half shows a unique numerical identifier; this can be used to distinguish components derived from the same template, e.g. gunzip:2 and gunzip:6.

The workflow as constructed in Wildfire is shown in Fig. 4, and its implementation without Wildfire is described in detail in [14]. The atomic component exonx is a program developed in-house to extract and store exons from a genbank file in fasta format; dice is a perl script used to break up a fasta file into smaller pieces. The noop.sh component is required to instruct GEL to copy the input files into the working directory. (The initial copying of input files will likely be an implicit feature of workflows in future versions of Wildfire, and so the explicit noop. sh component will no longer be necessary.) The remaining components (GNU gunzip, NCBI BLAST formatdb and blastall) are standard applications which we have incorporated as atomic components using our ACD editor.

The whole workflow takes less than 6000 seconds to run on a 128 CPU Pentium III cluster, whereas a sequential version of the same workflow required almost nine times longer. Profiling of the workflow shows that breaking up the 24 files of exons more evenly would significantly improve performance. Since all programs are run via the scheduler on the cluster, the workflow follows whatever scheduling policies are configured at the component-level. Hence, the workflow in its current form is already efficient from the point of view of resource use.

### Swarm optimisation

Real-life optimisation problems are often intractable and heuristics are the only choice for finding near optimal solutions. Particle Swarm Optimisation [15] is such a heuristic based on simulation of information exchange between leaders and followers observed in, for example, bird flocking.

The algorithm simulates individuals flying through the search space. On each iteration, the individuals are separated into a set of leaders and a set of followers, based on their fitness. The followers use the locations of the leaders to change their flying direction, i.e. search velocity. The location of each individual is computed based on its current location and flying direction. The new location is used to rank the fitness of individuals and subsequently the leader and follower sets.

Note that efficient Swarm Optimisation implementations exhibit both (1) *iteration *and (2) *parallelism: *successive generations must be simulated until a termination condition is met, and simulation of each generation entails simulation of many independent individuals. Therefore, a workflow tool suitable for implementing such algorithms must support iteration and parallelism.

**Figure 5:**
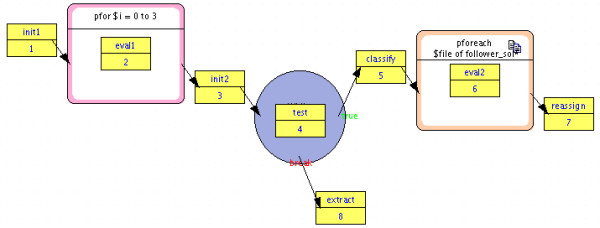
**Swarm optimisation example**. Swarm optimisation example. Component eval1 is executed four times in parallel within a parallel for loop. The circle denotes a while loop, with test as loop guard: if test returns false, then we follow the bottom branch to extract, otherwise we follow the right branch and test test again after reassign. Component eval2 is executed in parallel once for each file matching follower_sol*.

The workflow in Fig. 5 is a simplified implementation of a swarm algorithm by Ray et al. [16] implemented as a workflow. The algorithm is applied to a parameter estimation problem for a biochemical pathway model consisting of 36 unknowns and eight ordinary differential equations [17]. Components init1, eval1 and init2 are used to initialise and rank the individuals. Component test determines whether the workflow should terminate and extract collects together the results on termination of the simulation. Component eva12 is used to evaluate the fitness of an individual; note the outer parallel loop evaluates the fitness of each follower. The remaining components are used to select the leaders and followers.

Component test depends on both init2 and reassign; on the first iteration, test can start executing only after init2 has terminated, and on subsequent iterations, only after reassign has terminated. Since reassign itself hereditarily depends on test (i.e. reassign depends on eval2 which depends on classify which itself depends on test) we see there is a cyclic dependency. The while loop in GEL allows such dependencies and so is crucial for this workflow.

### Allergenicity prediction

Allergenicity prediction is the process of determining whether a new protein sequence is an allergen or not. Proteins known to induce allergic responses have been documented in allergen databases. One approach to allergenicity prediction is to determine, automatically, motifs from sequences in such a database, and then search for these motifs in the query sequences.

**Figure 6:**
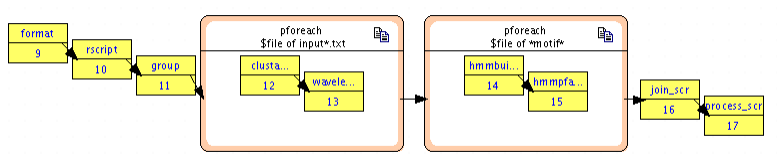
**An allergenicity prediction workflow**. The allergenicity prediction workflow from [23] constructed in Wildfire. The components in this workflow are all custom applications, or custom scripts calling standard applications. Components format, group, join_scr and process_scr are administrative programs which translate and convert files from one format to another. Component rscript uses the R-project to cluster the amino acid sequences from a database of known allergens. For each cluster, we align its sequences and use a wavelet algorithm to predict motifs. The resulting motifs are used to construct HMM profiles using hmmbuild. Finally, we use these profiles with hmmpfam to predict allergenic sequences. Components join_scr and process_scr collate and summarise the results.

The objective of the workflow in Fig. 6 is to test the accuracy of the approach described above, where protein sequence motifs are identified using an algorithm [18] based on wavelet analysis. From a group of 817 sequences, known to be allergens, we take a *learning *set consisting of a randomly selected subset covering 90% to be used for identification of motifs. The remaining 10% are used as query sequences for allegenicity prediction. We use the predictions to assess the accuracy of this approach.

Initially, we use ClustalW to generate the pairwise global alignment distances between the protein sequences in the learning set. We then use these distances to cluster the protein sequences by partitioning around medoids using the R project [19]. We use ClustalW again to align each cluster of protein sequences and we use the wavelet analysis algorithm on each aligned cluster to identify motifs in the protein sequences. For each identified motif, we build an HMM profile [20,21] in parallel. Note that the number of motifs is not know a priori; it can only be known at run-time. The HMM profiles are then used to search for the motifs in each query sequence, and thus predict whether it is an allergen or not. The accuracy of the predictions is computed to assess the effectiveness of this approach.

## Discussion

Bioinformaticians can use Wildfire as an integrated environment to *construct *and to *execute *workflows. The graphical user interface elements ease workflow construction by hiding the syntax of scripting languages. The constructed workflows can be executed across multiple processors (i) in the same server, (ii) in a cluster, or (iii) across several supercomputers across the Grid.

Wildfire is preconfigured to allow applications from EMBOSS to be used as workflow components. The user can add his own applications to be used as components by creating the necessary ACD files. Wildfire also hides this syntax by providing a wizard for creating and editing ACD files.

In a typical scenario, the user develops a workflow by visually constructing and executing small examples on his desktop or laptop. When the workflow is ready, the user can run it on real data on a shared resource such as a compute cluster running a scheduler. GEL workflows, such as those constructed in Wildfire, run well on shared resources since the components of the workflow are run through the scheduler. This gives the scheduler more opportunities to schedule the components with respect to whatever policies are configured: for example, a *fair share *policy would allow jobs from other users to run even when the workflow would otherwise monopolise the whole cluster.

We have described three applications of Wildfire in three different fields, and welcome readers to try Wildfire for themselves and solicit recommendations for improvements.

## Availability and requirements

### Wildfire

Wildfire is run on the client computer and allows the user to visually construct workflows. Wildfire invokes GEL (see below) to execute workflows. EMBOSS 2.8.0 [22] is required (2.9.0 is not yet supported) to run EMBOSS workflows.

**Availability:**

**Operating systems:** Platform Independent (tested on Windows, i386 Linux)

**Programming Language:** Java

**Other requirements:** Java 1.4.2, GEL

**Licence:** GPL

Currently, stand-alone mode is not available for Windows.

### GEL

GEL is an interpretor which executes GEL scripts generated by Wildfire. Currently only UNIX platforms are supported. GEL supports LSF, SGE and PBS clusters, SMP servers and Condor-based Grids.

**Availability:** 

**Operating systems:** GNU-style UNIX (tested on i386 Linux, ia64 Linux, Spare Solaris, Alpha Tru64)

**Programming Language:** Java

**Other requirements:** Java 1.4.2, bash

**Licence:** free for non commercial use, see 

## Authors' contributions

FT lead and coordinated the software engineering aspects of the project, and drafted this manuscript. FT and CCL co-designed Wildfire and GEL. CCL and HLY programmed, tested and debugged the software. LYP designed the tissue-specific transcript analysis workflow. PI and AK designed the allergen prediction workflow. AK participated in the concept, design and testing of the software and contributed to successive revisions of this manuscript. All authors read and approved the manuscript.
